# Multiple domains in the Crumbs Homolog 2a (Crb2a) protein are required for regulating rod photoreceptor size

**DOI:** 10.1186/1471-2121-11-60

**Published:** 2010-07-29

**Authors:** Ya-Chu Hsu, Abbie M Jensen

**Affiliations:** 1 Department of Biology, University of Massachusetts, Amherst, MA, 01003, USA; 2 Molecular and Cellular Biology Program, University of Massachusetts, Amherst, MA, 01003, USA; 3 Department of Ophthalmology, Margaret M. Dyson Research Institute, Weill Cornell Medical College, NYC, NY, 10021, USA

## Abstract

**Background:**

Vertebrate retinal photoreceptors are morphologically complex cells that have two apical regions, the inner segment and the outer segment. The outer segment is a modified cilium and is continuously regenerated throughout life. The molecular and cellular mechanisms that underlie vertebrate photoreceptor morphogenesis and the maintenance of the outer segment are largely unknown. The Crumbs (Crb) complex is a key regulator of apical membrane identity and size in epithelia and in Drosophila photoreceptors. Mutations in the human gene *CRUMBS HOMOLOG 1 *(*CRB1*) are associated with early and severe vision loss. Drosophila Crumbs and vertebrate Crb1 and Crumbs homolog 2 (Crb2) proteins are structurally similar, all are single pass transmembrane proteins with a large extracellular domain containing multiple laminin- and EGF-like repeats and a small intracellular domain containing a FERM-binding domain and a PDZ-binding domain. In order to begin to understand the role of the Crb family of proteins in vertebrate photoreceptors we generated stable transgenic zebrafish in which rod photoreceptors overexpress full-length Crb2a protein and several other Crb2a constructs engineered to lack specific domains.

**Results:**

We examined the localization of Crb2a constructs and their effects on rod morphology. We found that only the full-length Crb2a protein approximated the normal localization of Crb2a protein apical to adherens junctions in the photoreceptor inner segment. Several Crb2a construct proteins localized abnormally to the outer segment and one construct localized abnormally to the cell body. Overexpression of full-length Crb2a greatly increased inner segment size while expression of several other constructs increased outer segment size.

**Conclusions:**

Our observations suggest that particular domains in Crb2a regulate its localization and thus may regulate its regionalized function. Our results also suggest that the PDZ-binding domain in Crb2a might bring a protein(s) into the Crb complex that alters the function of the FERM-binding domain.

## Background

Vertebrate photoreceptors are morphologically complex and highly compartmentalized cells with large apical domains. The apical domain consists of a proximal inner segment and a distal outer segment, which is a modified cilium packed with intramembranous discs containing the photon-capturing opsins. Photoreceptors are not renewable like many other cell types, such as skin and intestinal cells, but each individual photoreceptor has the remarkable, and perhaps unique, ability to shed and renew a part of themselves- the outer segment. Photoreceptors shed the tips of their outer segments, which are then phagocytosed by the neighboring retinal pigmented epithelium [1,2]. It has been estimated that 10% of the rod outer segment is shed each day [3]. Remarkably little is known about the molecular and cellular mechanisms that control vertebrate photoreceptor morphogenesis and even less about how photoreceptors control the size of their outer segments through the processes of shedding and renewal.

Crumbs proteins are regulators of apical identity and size [4-6]. Previously we showed that the FERM protein Mosaic eyes (Moe; known as Epb4.1L5 in mammals and Yurt in Drosophila) is a novel component of the Crumbs complex and that loss-of-*moe *function results in an expanded apical domain, the outer segment, in rods [7,8]. Our observations led us to suggest that Crumbs may be a part of the outer segment renewal machinery in photoreceptors. Mutations in one of the three human *crumbs*-like genes, *CRB1*, cause severe and early onset vision loss diseases [9-12]. Missense mutations in *CRB1 *associated with disease are found in all domains, suggesting that all domains, including the extracellular and intracellular domains, contribute to CRB1 function (for review see [13]). Drosophila Crumbs is required for normal photoreceptor morphogenesis and the role of particular domains in Crumbs in photoreceptor morphogenesis has been examined [5,14,15]. The Crumbs homologs expressed in vertebrate photoreceptors share structural homology with Drosophila Crumbs [6,10,16]. The role of particular domains in vertebrate Crumbs homolog 2 (Crb2) proteins in regulating vertebrate photoreceptor morphogenesis has not been examined. To determine the function of specific domains in Crb2 proteins in vertebrate photoreceptors we generated transgenic zebrafish lines in which rods overexpress particular domains of the Crb2a protein. Our analyses suggest that multiple domains in Crb2a are important for protein localization and for its function in regulating the size of apical cellular compartments.

## Results

**Figure 1:**
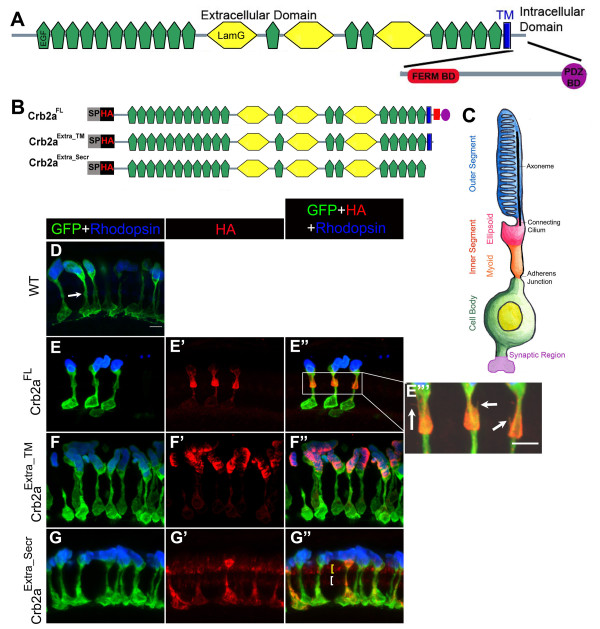
**Localization of Crb2a extracellular domain-containing constructs in rod photoreceptors and transgenic rod morphology**. Domain structure of zebrafish Crb2a protein. Crb2a is a single-pass transmembrane (blue) protein. The extracellular domain contains 19 EGF-like domains (green) and 3 laminin-like domains (yellow). The small intracellular domain contains two protein-protein interacting domains, the FERM-binding domain (FBD; red) and the PDZ-binding domain (PBD; purple). (B) Crb2a constructs containing the extracellular domain. All constructs have a signal peptide (SP) followed by an HA tag. Crb2a^Extra_TM ^has the same sequence as Crb2a^FL ^except the intracellular domain was deleted. Crb2a^Extra_Secr ^has only the extracellular domain. (C) The rod photoreceptor has four morphologically and functionally distinct compartments: two basal compartments, the cell body (green) and synaptic region (purple), and two apical compartments, the inner segment (orange and pink) and outer segment (blue) filled with membranous discs packed with Rhodopsin. The inner segment is further subdivided into the myoid region (orange) and ellipsoid region (pink). The apical and basal compartments are separated by an adherens junction. (D-G") Confocal z-projections of 6 d rods labeled with GFP (green), Rhodopsin (blue) and anti-HA (red) antibodies. (D) Wild-type rods. The adherens junction, the outer limiting membrane (OLM), is indicated by the white arrow. (E-E'") Crb2a^FL ^transgenic rods. Higher magnification of the inner segment showing fine processes (indicated by arrows) emerging from the inner segment (E"'). (F-F") Crb2a^Extra_TM ^transgenic rods. (G-G") Crb2a^Extra_Secr ^transgenic rods. Two rows of Crb2a^Extra_Secr ^are indicated by yellow and white brackets, Scale bar, 5 μm (D-G").

**Figure 2:**
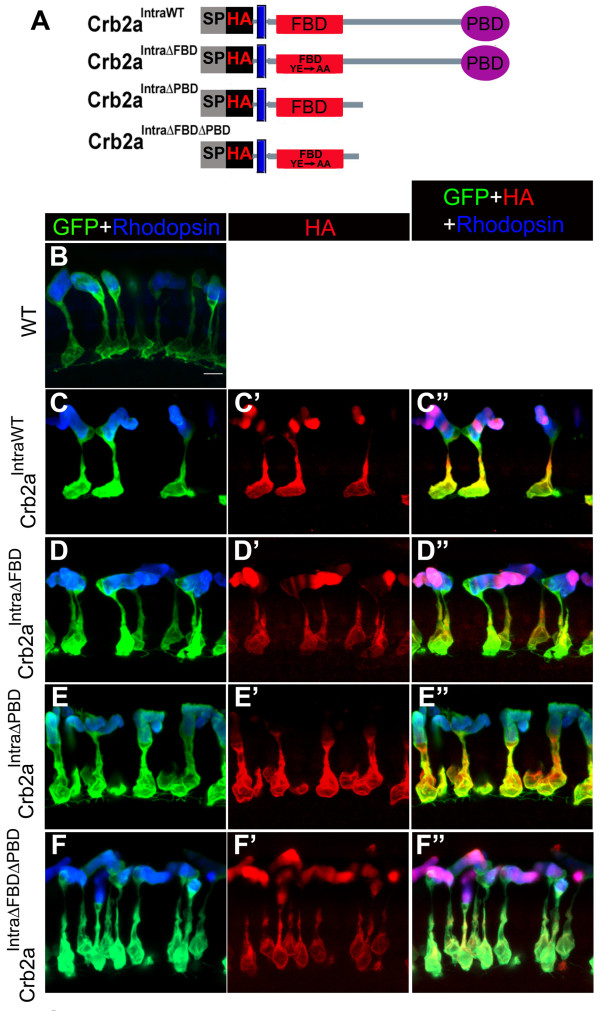
**Localization of Crb2a constructs lacking the extracellular domain in rod photoreceptors and transgenic rod morphology**. (A) Crb2a constructs lacking the extracellular domain. All constructs have a signal peptide (SP) followed by an HA tag. Crb2a^IntraWT ^contains the transmembrane domain and intracellular sequence of Crb2a. Crb2a^IntraΔFBD ^bears two mutations (Y10A and E16A) within the FERM-binding domain (FBD). Crb2a^IntraΔPBD ^has the sequence following the FBD that contains the PDZ-binding domain (PBD) deleted. Crb2a^IntraΔFBDΔPBD ^bears Y10A and E16A mutations within the FBD and the PBD the sequence following the FBD that contains the PDZ-binding domain (PBD) deleted. (B-F") Confocal z-projections of 6 d rods labeled with GFP (green), Rhodopsin (blue) and anti-HA (red) antibodies. (B) Wild-type rods. (C-C") Crb2a^IntraWT ^transgenic rods. (D-D") Crb2a^IntraΔFBD ^transgenic rods. (E-E") Crb2a^IntraΔPBD ^transgenic rods. (F-F") Crb2a^IntraΔFBDΔPBD ^transgenic rods. Scale bar, 5 μm (B-F").

In this study we investigated the contribution of specific domains of zebrafish Crb2a to its localization and function in regulating photoreceptor morphology. Zebrafish photoreceptors express two paralogous *crb2 *genes, *crb2a *and *crb2b*, but *crb2b *expression is rapidly down-regulated during differentiation [6,8]. Because *crb2a *expression is maintained in photoreceptors, we generated our constructs using the *crb2a *gene. We generated stable transgenic lines that overexpress seven different HA-tagged Crb2a constructs (see Figs. 1B and 2A) in zebrafish rods under the control of the Xenopus *rod opsin *promoter [17], which drives high levels of transgene expression specifically in rods soon after they become post-mitotic. We observed initial transgene expression around 2.5 days postfertilization (d) in rods before outer segment formation; expression was extensive by 3 d and continued through adulthood (data not shown). The Crb2a constructs form two groups, those that contain the extracellular domain (Fig. 1B), and those that lack the extracellular domain of Crb2a (Fig. 2A). To generate stable transgenic lines, we used the Tol2 transposon method [18,19]. We generated three independent lines for each of the constructs because we did not know at the beginning of the study whether the site of insertion would affect transgene expression levels. The three lines generated for each construct showed generally similar levels of transgene product and thus, we present data from one line for each construct.

### Localization of extracellular *crb2a *transgene products in rods

The role of the extracellular domain in vertebrate Crb1 and Crb2 proteins is unknown and no molecules have been identified that interact with this region. In Drosophila, the extracellular domain plays a critical role in determining the length of the stalk region in photoreceptors [5]. The structure of zebrafish Crb2a protein is similar to Drosophila Crumbs and has a large extracellular domain with 19 EGF-like domains and 3 Laminin-like domains, a single pass transmembrane domain, and a small intracellular domain with a FERM binding domain (FBD) and a PDZ-binding domain (PBD; Fig. 1A). We asked whether the role of Crb2a extracellular domain is conserved across species and sought to determine the role of the extracellular domain of Crb2a in protein localization and rod morphogenesis. We generated transgenics expressing the full-length Crb2a protein (Crb2a^FL^), the extracellular domain of Crb2a containing the transmembrane domain (Crb2a^Extra_TM^), and the extracellular domain of Crb2a that lacks the transmembrane domain and, thus, is a secreted Crb2a extracellular domain (Crb2a^Extra_Secr^; Fig. 1B),

The rod photoreceptor has four morphologically and functionally distinct compartments. There are two basal compartments, the proximal cell body and the distal synaptic region, and two apical compartments, the proximal inner segment and the distal outer segment that is filled with membranous discs packed with photon-capturing Rhodopsin molecules (Fig. 1C). The apical and basal compartments are separated by a specialized adherens junction called the outer limiting membrane (OLM). The inner segment can be further subdivided into the proximal myoid region and the distal ellipsoid region (Fig. 1C). All the transgenic lines were made in or crossed into the Tg(*Xop:EGFP*) line [20] for analysis of rod morphology. The four compartments are visible in rods in the Tg(*Xop:EGFP*) background when labeled with anti-Rhodopsin antibodies [21], the OLM is also visible as a small gap or constriction in GFP fluorescence (Fig. 1D, arrow).

At 6 d, which is about 3 days after the first rod birthdates, Crb2a^FL ^protein localized primarily to the inner segment, intensively in the proximal myoid and at a lower level in the ellipsoid, and very little was found on the plasma membrane of the cell body and none was found in the outer segment (Fig. 1E-E"'). The myoid region of the inner segment of Crb2a^FL ^expressing rods was enlarged (Fig. 1E-E"'). This morphology was so unique and distinct that we could easily distinguish Crb2a^FL ^rods from wild-type or other transgenic lines by observing GFP fluorescence alone. We also observed that ectopic processes often projected from the inner segments of Crb2a^FL ^expressing rods (Fig. 1E"').

Crb2a^Extra_TM ^was very strong in the outer segment where we observed very fine stripes of Crb2a^Extra_TM ^that appear more concentrated on one side of disks in the outer segment, perhaps near the axoneme, and there was weak plasma membrane labeling in the cell body and inner segment (Fig. 1F-F" and Additional file 1B, B'). Crb2a^Extra_Secr ^localized throughout the entire expressing rod except the outer segment (Fig. 1G-G"). We observed Crb2a^Extra_Secr ^in the region where Crumbs (predominantly Crb2a) proteins normally localize, just apical to the OLM (Fig. 1G", white bracket) and surrounding the inner segments of cones (Fig 1G", yellow bracket and Additional file 2). We observed no Crb2a^Extra_Secr ^below the OLM except within the cell bodies of the expressing rods. Single z-sections of Crb2a^FL^-, Crb2a^Extra_TM^- and Crb2a^Extra_Secr^-expressing rods are shown in Additional file 1A-C'.

### Localization of intracellular *crb2a *transgene proteins in rods

In order to investigate the role of the intracellular domain, consisting of the FERM-binding and PDZ-binding domains, we over-expressed several Crb2a constructs that lack the extracellular domain (Fig. 2A). We found that the localization of the Crb2a constructs that lack the extracellular domain is very different from those that retain the extracellular domain. At 6 d, Crb2a^IntraWT ^localized to the cell body plasma membrane, the proximal inner segment including the Golgi apparatus (see Additional file 3 for Golgi labeling in Crb2a transgenics), and outer segment (Fig. 2C-C"). Stripes of Crb2a^IntraWT ^were often observed in the outer segments (Fig. 2C') and the number of stripes correlated with the number of light cycles to which the cells had been exposed. The intracellular construct that lacks a functional FERM binding domain, Crb2a^IntraΔFBD^, localized similarly to Crb2a^IntraWT^, with labeling in the cell body plasma membrane, proximal inner segment (presumptive Golgi apparatus), and outer segment (Fig. 2D-D"). The amount of Crb2a^IntraΔFBD ^protein in the cell body plasma membrane appeared lower than Crb2a^IntraWT^. We also observed stripe patterns of Crb2a^IntraΔFBD ^in the outer segments (Fig. 2D'), suggesting that this construct may be regulated similarly to Crb2a^IntraWT^. The intracellular construct that lacks the PDZ binding domain, Crb2a^IntraΔPBD^, localized mostly to the plasma membrane of cell body and the inner segment and there was very little labeling in the outer segment (Fig. 2E-E"). We also made a control construct that lacks a functional FERM binding domain and PDZ binding domain, Crb2a^IntraΔFBDΔPBD^, and found this construct localized to the plasma membrane of the cell body, proximal inner segment (presumptive Golgi apparatus), and outer segment (Fig. 2F-F"), similar to Crb2a^IntraWT ^and Crb2a^IntraΔFBD^. Single z-sections of Crb2a^IntraWT^-, Crb2a^IntraΔFBD^-, Crb2a^IntraΔPBD^- and Crb2a^IntraΔFBDΔPBD^-expressing rods are shown in Additional file 1D-G'. We note that the levels of Crb2a^IntraWT^, Crb2a^IntraΔFBD ^and Crb2a^IntraΔFBDΔFBD ^in the cell bodies are much lower than Crb2a^IntraΔPBD^, which may not be clear from the confocal images (Fig. 2). The only intracellular construct that did not localize to the outer segment was Crb2a^IntraΔPBD^, suggesting that this construct may be actively retained in the inner segment and cell body by the remaining FBD or that this construct is trafficked differently than the other constructs.

### Mutations in the FERM binding domain of Crb2a (Crb2a^IntraΔFBD^) abolish in vitro binding to the FERM protein Moe

**Figure 3:**
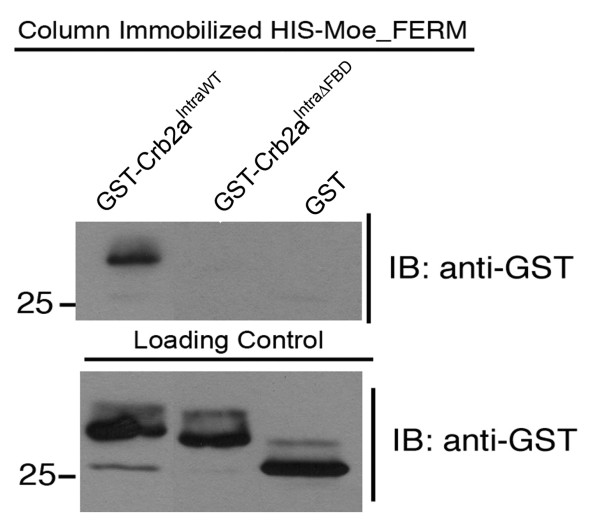
**The FBD mutations Crb2a abolish binding to the FERM protein Moe**. In vitro GST interaction between HIS-Moe_FERM immobilized on the column with GST-Crb2a^IntraWT ^and GST-Crb2a^IntraΔFBD^. Loading control showing 1/40 input blotted and labeled with anti-GST.

Studies in Drosophila assumed that substitution of two key residues in the FBD would compromise the binding between the FERM-binding domain in Crumbs and the FERM protein, but this idea was not directly tested [14]. In this study we tested whether the two mutations, Tyr10 to Ala and Glu16 to Ala (Fig. 2A), in the FBD are sufficient to abolish the interaction between Crb2a and the FERM protein Moe. We generated GST tagged Crb2a^IntraWT ^and Crb2a^IntraΔFBD ^and tested their ability to bind the His-tagged FERM domain of Moe. We found that recombinant HIS-Moe_FERM bound to GST-Crb2a^IntraWT ^but very poorly to GST-Crb2a^IntraΔFBD^, at a level close to the negative control of GST alone (Fig. 3). This result suggested that the two mutations we made to generate Crb2a^IntraΔFBD ^were sufficient to interfere with Moe-Crb2a interactions. This suggests that overexpressed Crb2a^IntraΔFBD ^may lose its binding to Moe and thus becomes mislocalized to the outer segment (Fig. 2D').

### Localization of Rhodopsin is unaffected in transgenic rods

Since many of our Crb2a transgene products are mislocalized to the outer segment and cell body, we asked whether over-expression of any of the Crb2a constructs interferes with Rhodopsin localization in the outer segment. To recognize Rhodopsin we used a Rhodopsin-specific monoclonal antibody (clone R6-5 [21]). In wild-type rods at 6 d, Rhodopsin localized only to the outer segment (Fig. 1B, 2B), and we found that Rhodopsin localization is normal in all our transgenic lines (Figs. 1 and 2 and data not shown), indicating that over-expression of either the intracellular or extracellular domains of Crb2a does not interfere with Rhodopsin transport or targeting even though many transgene products localized to the outer segment.

### Overexpression of the Crb2a intracellular domain increases outer segment size

**Figure 4:**
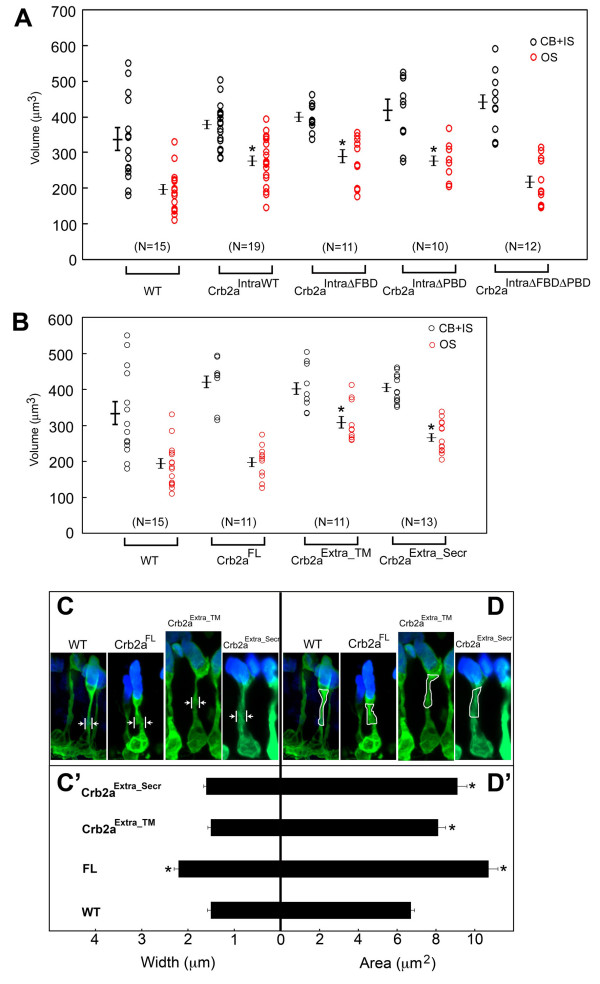
**Measurement of rod size in Crb2a transgenics**. (A) The average combined volume of rod cell body and inner segment (black circles) measured 332 ± 30 μm^3 ^(wildtype), 372 ± 13.6 μm^3 ^(Crb2a^IntraWT^), 394 ± 12.2 μm^3 ^(Crb2a^IntraΔFBD^), 416 ± 28.9 μm^3 ^(Crb2a^IntraΔPBD^), and 436 ± 22.8 μm^3 ^(Crb2a^IntraΔFBDΔPBD^). The average volume of a rod outer segment (red circles) measured 190 ± 15.7 μm^3 ^(wildtypes), 277 ± 15.4 μm^3 ^(Crb2a^IntraWT^), 283 ± 20.6 μm^3 ^(Crb2a^IntraΔFBD^), 278 ± 15.9 μm^3 ^(Crb2a^IntraΔPBD^), and 212 ± 18.9 μm^3 ^(Crb2a^IntraΔFBDΔPBD^). (B) The average combined volume of rod cell body and inner segment (black circles) measured 332 ± 30 μm^3 ^(wildtype), 426 ± 17.5 μm^3 ^(Crb2a^FL^), 401 ± 17.8 μm^3 ^(Crb2a^Extra_TM^), and 405 ± 11 μm^3 ^(Crb2a^Extra_Secr^). The average volume of a rod outer segment (red circles) measured 190 ± 15.7 μm^3 ^(wildtype), 198 ± 13.8 μm^3 ^(Crb2a^FL^), 316 ± 17.2 μm^3 ^(Crb2a^Extra_TM^), and 268 ± 12.1 μm^3 ^(Crb2a^Extra_Secr^). (C, C') Width of the inner segment myoid region was marked as in (C) and the measurements are shown in (C'). The width of the myoid region is significantly larger in Crb2a^FL ^rods (2.2 ± 0.1 μm) than wildtypes (1.5 ± 0.08 μm). Crb2a^Extra_TM ^(1.5 ± 0.07 μm) and Crb2a^Extra_Secr ^(1.6 ± 0.06 μm) are similar to wildtypes. (D, D') Area of the myoid region was outlined as in (D) and the measurements are shown in (D'). The area of the myoid region is significantly larger in Crb2a^FL ^(10.7 ± 0.5 μm^2^), Crb2a^Extra_TM ^(8.1 ± 0.4 μm^2^), and Crb2a^Extra_Secr ^(9.1 ± 0.5 μm^2^) compared to wildtypes (6.7 ± 0.2 μm^2^). Each circle represents a measurement of a single rod. Shown values are mean ± s.e.m, *, *p *< 0.005, student's *t *test.

Our previous work suggested Moe is a negative regulator of the Crumbs function in photoreceptors, and loss-of-*moe *function resulted in a larger outer segment and affected photoreceptor morphology [8]. We sought to determine whether overexpression of any of the Crb2a constructs affected rod size or morphology. We examined rod morphology at 6 d in the lines that express the intracellular domains of Crb2a. Gross morphology of rods as visualized by GFP in combination with anti-Rhodopsin antibody labeling was normal in Crb2a^IntraWT^, Crb2a^IntraΔFBD^, Crb2a^IntraΔPBD ^and Crb2a^IntraΔFBDΔPBD ^transgenics (Fig. 2A-F). However, when we measured the volume or size of rod outer segments at 6 d, we found that the volume of outer segments of Crb2a^IntraWT^, Crb2a^IntraΔFBD^, Crb2a^IntraΔPBD ^expressing rods increased significantly compared to wildtypes (Fig. 4A, red circles), whereas, the size of the inner segment plus cell body was not significantly different from wildtypes (Fig. 4A, black circles). These results suggest that over-expression of any defined domain of the Crb2a intracellular domain increases the size of outer segments in rods without affecting gross morphology.

### Overexpression of the Crb2a extracellular domain increases the size of inner and outer segments

We measured the size of Crb2a^FL^, Crb2a^Extra_TM ^and Crb2a^Extra_Secr ^expressing rods at 6 d. The outer segments of Crb2a^FL ^rods were not significantly larger than wild-type rods but outer segments of Crb2a^Extra_TM ^and Crb2a^Extra_Secr ^rods are larger than those in wildtypes (Fig. 4B). The most dramatic morphological change we observed was in Crb2a^FL ^rods, where the myoid region of the inner segment was enlarged (Fig. 1E-E"). The enlargement of the myoid in Crb2a^FL ^rods was most obvious in the area immediately above the OLM, therefore we measured the width the myoid just apical to the OLM in confocal z-projections as indicated in Fig. 4C. The width of the myoid in Crb2a^FL ^expressing rods was nearly 50% wider than wildtypes (Fig. 4C, C'). The widths of the myoid in Crb2a^Extra_TM ^or Crb2a^Extra_Secr ^rods were not significantly different from wildtypes (Fig. 4C, C'). We also quantified the size of the inner segment by measuring the area of the entire myoid in confocal z-projections as indicated in Fig. 4D. This measurement showed that Crb2a^FL^, Crb2a^Extra_TM^, Crb2a^Extra_Secr ^rods had a significant increase in the size of myoid (Fig. 4D').

### Monoclonal antibody zs-4 recognizes the extracellular domain of Crb2a

Our next goal was to determine whether expression of any of our transgenes affected the levels or localization of endogenously expressed Crb2a/b proteins. The panCrb antibodies that we used previously and the one we raised and used in this study were generated against a C' terminal peptide that includes the PBD that is highly conserved in all members of the Crumbs family of proteins and these antibodies recognize all zebrafish Crumbs proteins [8] (and data not shown). Thus, the panCrb antibody recognizes all constructs that retain the C' terminal peptide and cannot be used to distinguish between those constructs and endogenous Crumbs proteins. In order to distinguish between endogenous Crb2a/b proteins and intracellular transgene products, we required an antibody that recognizes the extracellular domain of Crb2a.

**Figure 5:**
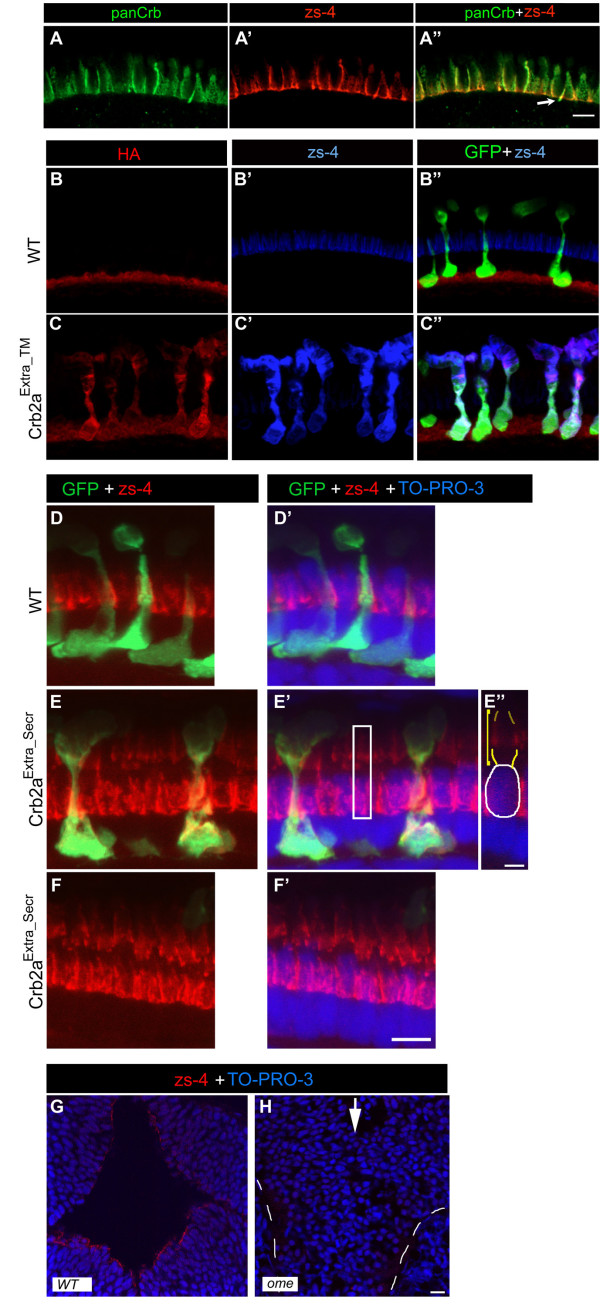
**The zs-4 antibody recognizes the extracellular domain of Crb2a**. (A-A") Single confocal z-section of the photoreceptor layer in a 6 d wild-type retina labeled with rabbit panCrb antibodies (A) and zs-4 monoclonal (A') and the two channels merged (A"). The arrow indicates where anti-panCrb and zs-4 labeling does not appear to fully overlap. (B-C") Confocal z-projection of a 6 d retina labeled with anti-HA antibody (monoclonal IgG_3_; red) and zs-4 antibody (blue) and merged with anti-GFP (green). (B-B") Wild-type retina. (C-C") Crb2a^Extra_TM ^transgenic retina. Endogenous Crb2a labeling is weak in comparison to Crb2a^Extra_TM ^protein (C"). (D-F') Confocal z-projection of the photoreceptor layer in a 6 d retina labeled with zs-4 antibody (red), rods express GFP (green). TO-PRO-3 (blue) labeling reveals the position of nuclei in the photoreceptor layer. (D-D') Wild-type retina. (E-E") Crb2a^Extra_Secr ^transgenic retina. Single z-section from the white boxed area in E', showing zs-4 labeling around the inner segment (indicated by yellow bracket and outlined in yellow) of a double-cone, the cell-body is circled in white (E"). (F-F') Crb2a^Extra_Secr ^transgenic retina where no rods are present. (G-H) Single z-section of the brain ventricular region of a 40 hour postfertilization embryo labeled with zs-4 antibody (red) showing ventricular surface labeling. Cell nuclei are labeled with TO-PRO-3 (blue). (G) Wild-type embryo. (H) *ome *(*crb2a*) mutant embryo. The midline (where the ventricles would normally form) is indicated by the white arrow, and eyes indicated by dashed white lines. Scale bars, 5 μm (A-A"', D-F') 10 μm (G, H).

In a search for such an antibody, we tested the monoclonal antibody zs-4 and found that its labeling pattern is remarkably similar to panCrb antibody labeling in the photoreceptor layer. We performed double labeling with zs-4 and panCrb antibodies and found a nearly identical localization of the two antibodies (Fig. 5A-A"). They do not colocalize perfectly (Fig. 5A", arrow), suggesting that zs-4 labels only Crb2a or only Crb2b [8]. Since *crb2b *is rapidly down-regulated and most of the panCrb labeling is also labeled by zs-4, it was more likely that zs-4 recognizes Crb2a. Unfortunately, zs-4 does not recognize protein in western blot (data not shown). However, using the Crb2a^Extra_TM ^and Crb2a^Extra_Secr ^transgenics that we produced, we show that zs-4 recognizes the extracellular domain of Crb2a. To show that zs-4 recognizes the extracellular domain of Crb2a, we labeled retinal sections from two of our transgenic lines, Crb2a^Extra_TM ^and Crb2a^Extra_Secr^, with the zs-4 antibody and an anti-HA antibody. Since zs-4 and the anti-HA (clone 16B12) are both IgG_1 _isotype, we tested several other anti-HA polyclonals and monoclonals that might be compatible for zs-4 double-labeling. The best anti-HA antibody we tested was an IgG_3 _isotype but it was weaker than clone 16B12 and the background is higher (Fig. 5B, C). Nonetheless, we found that zs-4 labeling resembled the anti-HA labeling except that, in addition, it also labels the endogenous Crumbs proteins localized to the inner segment region (Fig. 5B'-C' and data not shown). The zs-4 antibody also recognizes Crb2a^Extra_Secr ^that localizes around the inner segments of double cones (Fig. 5E-F' and Additional file 2). We also observed that zs-4 labeling was lost in *ome *(*crb2a*) mutant embryos (Fig. 5G, H) and zs-4 did not label tissues that express only *crb2b*, such as kidney podocytes (YCH and AMJ, unpublished observation). Our observations, taken together, support the conclusion that zs-4 recognizes the extracellular domain of Crb2a, and, thus, this antibody was a useful tool in this study to localize the endogenous Crb2a proteins in Crb2a^Intra ^transgenics.

### Expression of Crb2a^FL ^may alter the localization of endogenous Crumbs proteins

**Figure 6:**
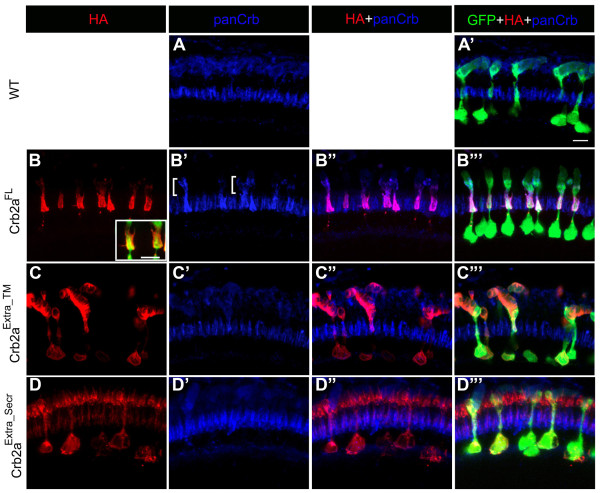
**Effects of Crb2a extracellular domain-containing constructs on endogenous Crumbs proteins**. (A-D"') Confocal z-projections of 6 d rods labeled with GFP (green), panCrb (blue) and anti-HA (red) antibodies. (A, A') Wild-type rods. (B-B"') Crb2a^FL ^transgenic rods. Inset in B shows fine processes emerging from the inner segment. Bracket in B' indicates a region of strong panCrb labeling that is weakly labeled by anti-HA antibodies. (C-C"') Crb2a^Extra_TM ^transgenic rods. (D-D"') Crb2a^Extra_Secr ^transgenic rods. Scale bar, 5 μm.

Constructs similar to some of ours were tested for dominant affects in Drosophila, and the over-expression of some Crumbs constructs caused mislocalization of other apical polarity proteins, but it was not reported whether over-expression of Crumbs constructs affected the localization of endogenous Crumbs proteins [5,14]. To determine whether endogenous Crb2a/b proteins were mislocalized in the Crb2a^FL ^transgenics, we double labeled sections with anti-HA (clone 16B12) and anti-panCrb antibodies and examined whether the anti-HA and panCrb labeling appeared 100% coincident. This is an imperfect experiment because the relative affinities of the zs-4 monoclonal and the panCrb polyclonal are likely to be different but it is the best approximation available. In wildtypes, anti-panCrb labels primarily the inner segment region of photoreceptors with additional weak labeling in the outer segment region, which could be background (Fig. 6A). We observed that in Crb2a^FL ^expressing rods, anti-panCrb antibody labeling seems to extend beyond anti-HA labeling, and labels the distal myoid and ellipsoid regions of the rod inner segments (Fig. 6B', brackets). The inset in Fig. 6B shows the ectopic inner segment processes in Crb2a^FL ^transgenic rods. Because both Crb2a^Extra_TM ^and Crb2a^Extra_Secr ^transgene products lack the intracellular domain, we used anti-HA to localize transgene products and anti-panCrb to localize endogenous Crb2a/b proteins. We did not observe mislocalization of anti-panCrb antibody labeling in Crb2a^Extra_TM ^or Crb2a^Extra_Secr ^transgenics (Fig. 6C-C"' and 6D-D"').

### Overexpression of intracellular Crb2a constructs does not alter localization of endogenous Crb2a proteins, Prkci or Moe

**Figure 7:**
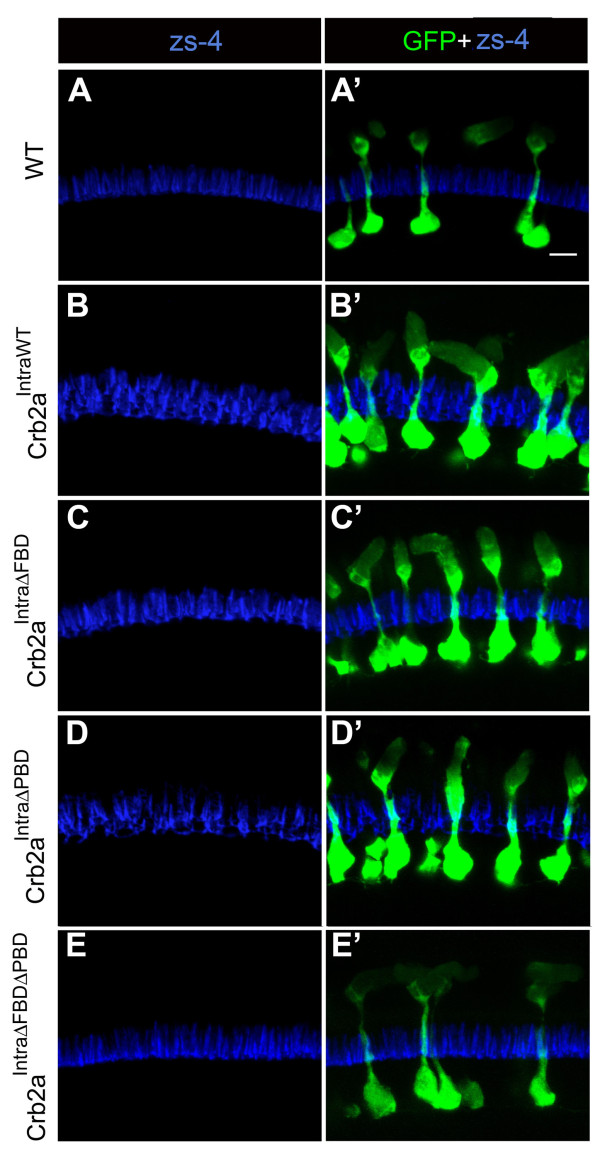
**Effects of Crb2a constructs that lack the extracellular domain on endogenous Crb2a**. (A-E') Confocal z-projections of 6 d rods labeled with zs-4 (blue) and anti-GFP (green) antibodies. (A, A') Wild-type rods. (B, B') Crb2a^IntraWT ^transgenic rods. (C, C') Crb2a^IntraΔFBD ^transgenic rods. (D, D') Crb2a^IntraΔPBD ^transgenic rods. (E, E') Crb2a^IntraΔFBDΔPBD ^transgenic rods. Scale bar, 5 μm.

To address whether expression of the intracellular constructs caused mislocalization of endogenous Crb2a, we used zs-4 antibody to localize endogenous Crb2a in Crb2a^IntraWT^, Crb2a^IntraΔFBD^, Crb2a^IntraΔPBD ^and Crb2a^IntraΔFBDΔPBD ^transgenic lines (Fig. 7). We found that zs-4 labeling in Crb2a^IntraWT ^(Fig. 7B), Crb2a^IntraΔFBD ^(Fig. 7C), and Crb2a^IntraΔPBD ^(Fig. 7D) and Crb2a^IntraΔFBDΔnΔPBD ^(Fig. 7E) was similar to that in wildtypes (Fig. 7A), indicating that overexpression of these constructs did not alter endogenous Crb2a localization.

We asked whether overexpression of Crb2a constructs alters the localization of other Crumbs complex components such as Moe or those that interact indirectly, such as Prkci (also known as aPKCΔλ) [22-24]. We used antibodies against Moe and Prkci to localize these two proteins in our transgenics. We found that the localization of Moe and Prkci was unaffected in all transgenic lines (Additional file 4 and Additional file 5, respectively).

## Discussion

In this study we sought to determine which domains in Crb2a contribute to its localization and function in rods. Zebrafish rods express two *crb2 *paralogs, *crb2a *and *crb2b*, whereas *crb1 *expression was not detected in the retina [6,8]. Mouse rods also express two *crumbs *orthologs, *crb1 *and *crb2 *[8,25,26]. The observation that in zebrafish cells in the retina express two *crb2 *genes but no *crb1 *suggests that one or both of the *crb2 *genes has adopted the function of *crb1*. While mutations in human *CRB1 *are associated with several severe and early onset retinal degeneration diseases [9,10] (and reviewed in [27]), as yet no human disease has been associated with *CRB2 *mutations. Loss-of-*crb1 *function in mice also causes retinal defects but they appear less severe than those observed in humans; the OLM is disrupted in the *rd8 *mouse and inner and outer segments are smaller than normal [28,29]. We observed no defects in the OLM in any of the *crb2a *transgenic lines we created; anti-ZO-1 labeling was normal (data not shown) and adherens junctions in transgenic rods were visible as a distinct gap in GFP in the inner segment. As yet, loss-of-*crb2 *function in mice has not been reported, so its role in mammalian photoreceptors remains unknown.

Loss-of-*crb2b *function resulted in reduced photoreceptor apical size and we previously reported that loss-of-*moe *function, a putative negative regulator or Crumbs protein function, resulted in larger than normal outer segments [6,8,30]. In this study, overexpression of Crb2a^IntraWT^, Crb2a^IntraΔFBD^, Crb2a^IntraΔPBD^, Crb2a^Extra_TM^, or Crb2a^Extra_Secr ^resulted in a significant increase in outer segment size and without interfering with normal development of rods. This result supports our hypothesis that Crb2a may be involved in the renewal mechanism in photoreceptors. Overexpression of Crb2a^IntraWT^, Crb2a^IntraΔFBD^, Crb2a^IntraΔPBD ^proteins may increase outer segment size by competing for negative regulators of endogenous Crumbs proteins. For example, the FBD in Crb2a^IntraΔPBD ^may compete with endogenous Crb2a for binding to Moe, a suggested negative regulator of Crumbs protein function [6,8,30], and, thus, could lead to potentially more activity of endogenous Crb2a and a larger outer segment. It is more difficult to envision a mechanism by which overexpression of Crb2a^Extra_TM ^and Crb2a^Extra_Secr ^increases outer segment size without knowing what molecules interact with the extracellular domain of Crumbs proteins. Transgene expression likely increases outer segment size by increasing outer segment growth rather than decreasing outer segment shedding because at 6 d shedding has yet to begin; we see no RPE phagosomes at 6 d by immunocytochemistry or TEM (AMJ, unpublished observation).

Crb family proteins and several components of the Crumbs complex have a restricted localization just apical to adherens junctions in epithelia or the outer limiting membrane (OLM) in photoreceptors [6,8,28,29,31-33]. Crb family proteins are found in photoreceptor inner segments immediately apical to the OLM and in Müller glial microvillar processes that project into the inner segment region [8,32]. In zebrafish rods there is a morphologically distinct region just apical to the OLM that can be recognized as a bulge in the proximal inner segment (see Fig. 1D, arrow). In mouse, Crb2 localized by immunoEM to this region [32]. Our current findings suggest that domains in both the extracellular region and intracellular regions of Crb2a contribute to its proper localization in rods. Only Crb2a^FL ^localization in the myoid region approximated the normal localization of endogenous Crb2a/b proteins, although its localization was expanded and this expansion caused the bulge to expand. The functional significance of this region in photoreceptors is unknown.

Crb2a^IntraΔPBD ^was retained in the inner segment and cell body plasma membrane and very little was found in the outer segment in contrast to Crb2a^IntraΔFBD^, which localized mostly to the outer segment. These results suggest that the FBD is responsible for retaining Crb2a in the inner segment. Crb2a^IntraWT ^is also found in the outer segment, however, and it retains the FBD (and the PBD). Why? One possible explanation is that proteins brought into the Crb complex by the PBD disrupt or alter the interaction between the FBD and its binding partner and thus Crb2a^IntraWT ^behaves more like Crb2a^IntraΔFBD^. The PBD could bring PRKCi into the Crb complex, PRKCi could phosphorylate the FBD and thus lower its affinity for Moe, which localizes cortically in the inner segment and cell body [8], and, thus, Crb2a^IntraWT ^localizes similarly to Crb2a^IntraΔFBD ^and Crb2a^IntraΔFBDΔPBD^. Drosophila Crumbs has been shown to be a substrate for phosphorylation by aPKC (orthologue of PRKCi) and Crb activity during embryogenesis is regulated by phosphorylation [24]. Finally, the observation that several transgene products localize to the outer segment in the absence of any outer segment 'targeting signal' suggests that the outer segment could be the default localization for proteins that lack cytoskeletal (or extracellular) anchorage. Observations by Baker and colleagues lead them to also suggest that the rod outer segment seems to be the default localization for single-pass transmembrane proteins [34].

Crb2a^Extra_TM ^also localizes to the outer segment and this finding is different from that observed in fly photoreceptors where an equivalent construct localized to the stalk membrane [5]. We also found that Crb2a^Extra_TM ^expression in the outer segment is very different from Crb2a^IntraWT ^and Crb2a^IntraΔFBD^; Crb2a^Extra_TM ^protein forms fine stripes that are concentrated on one side of the outer segment, perhaps near the axoneme. We have no explanation of why Crb2a^Extra_TM ^is more concentrated on one side of the outer segment other then suggesting that since Crb2a^Extra_TM ^is a much larger protein than Crb2a^IntraWT^, Crb2a^IntraΔFBD ^and Crb2a^IntraΔFBDΔFBD ^and, consequently, it would be less likely to diffuse freely in the disk membrane.

The localization of Crb2a^Extra_Secr ^is intriguing. It is found in the cell body and in and around the inner segment of Crb2a^Extra_Secr ^rods as well as around the inner segments of neighboring cones. It is possible that the secreted Crb2a extracellular domain is trapped by an unknown receptor located on cone inner segments or Müller processes in the region. Functionally, overexpression of Crb2a^Extra_Secr ^in rods led to a small increase in the size of the outer segment and a modest increase in the area of the myoid region, which is opposite to the effect observed in Drosophila where overexpression of this construct shortened the stalk [5]. One possible explanation could be that most Crb2a^Extra_Secr ^protein is sequestered away from the site where it could interfere with normal Crb2a signaling at the base of the myoid in rods; most Crb2a^Extra_Secr ^protein localizes to the region near rod ellipsoids and cone inner segments.

While we cannot exclude entirely the possibility that differences in protein folding between the different constructs contribute to differences in localization, we think it is unlikely for the following reasons. First, given that the extracellular domain is identical in the three constructs that retain that domain (Crb2a^FL^, Crb2a^Extra_TM^, and Crb2a^Extra_Secr^) it seems unlikely that these proteins would fold differently in the ER. It is also unlikely that the presence of the intracellular domain would alter folding kinetics in the ER but we cannot exclude the possibility that it could affect time spent in the ER. Two, it seems unlikely that the small intracellular domain (37 amino acids at the longest) is subject to folding, given what is known about other FERM-binding domains and PDZ-binding domains. Third, the zs-4 antibody, which only recognizes the native (i.e. folded) extracellular domain of Crb2a, recognizes all constructs that retain the extracellular domain, suggesting that the extracellular domain is folded properly.

Overexpression of Crb2a^FL ^had the greatest effect on rod morphology and dramatically increased the width and area of the inner segment myoid region. Similarly, overexpression of full-length Crb had the greatest effect on stalk length in Drosophila photoreceptors [5]. It has been suggested that the inner segment of vertebrate photoreceptors may be a homologous structure to the stalk region in ommatidial photoreceptors in insects, as both lie in between the sensory compartment (outer segment in vertebrates and rhabdomere in insects) and the cell body [35]. The mechanism of inner segment enlargement in Crb2a^FL^-expressing rods remains unclear. It seems unlikely that it is due to enlargement of the endoplasmic reticulum as two other constructs (Crb2a^Extra_TM ^and Crb2a^Extra_Secr^) that are identical in the extracellular domain and expressed at similar levels do not produce such an enlargement (Additional file 6). It also seems that it is unlikely to be due to enlargement of the Golgi apparatus for similar reasons and we did not observe an enlarged Golgi apparatus in Crb2a^FL^expressing rods (Additional file 3). We also observed that overexpression of Crb2a^FL ^resulted in the appearance of fine processes emerging from the inner segment myoid. Interestingly, we also observed ectopic processes in the inner segment myoids of rods that lack Moe function, the FERM protein shown to bind the FBD of Crumbs proteins, and which was suggested to act as a negative regulator of Crumbs proteins [8,36]. The molecular origin of these processes remains unclear.

## Conclusions

The mechanism by which Crumbs proteins regulate apical cell polarity and apical membrane size remains mysterious. In Drosophila photoreceptors, both the extracellular and intracellular domains are important for Drosophila photoreceptor development and morphogenesis, in contrast, the extracellular domain seems largely dispensable for embryogenesis [4,5,15,37]. Our observations suggest that the functions of particular domains in Crb2a in regulating photoreceptor morphology are partly conserved with those in Drosophila Crb. Our results show that multiple domains in Crb2a are required for its location and function in rods. Since the extracellular domain of Crb2a is important for function, as in Drosophila photoreceptors [5], the identification of interactors that bind to the extracellular domain is especially important.

## Methods

### Animals

AB wild-type strain, Tg(*Xop:EGFP*);*alb*^-/+^, the Tg(*Xop:Crb2a*), *ome*^*m98 *^fish lines were maintained and staged as previously described according to Westerfield [38]. All experiments involving animals were performed with approval by and in accordance with the University of Massachusetts-Amherst Institutional Animal Care and Use Committee (IACUC).

### Cloning

Diagrams of the transgene constructs are shown in Figs. 1B and 2A. The constructs were cloned behind 0.8 kB of the Xenopus *rod opsin *promoter (*Xop*; [17]). All constructs have an N'terminal signal peptide (SP) taken from zebrafish Crb2b (SignalP 3.0 Server) and an influenza hemagglutinin (HA) tag (YPYDVPDYA) just after the SP. To make Crb2a^FL^, the SP of Crb2a was substituted with the Crb2b SP and the HA tag was engineered immediately after the predicted SP cleavage site. To make Crb2a^Extra_Secr ^and Crb2a^Extra_TM^, we used site-directed mutagenesis to place a stop codon just before and after the transmembrane domain, respectively. To make Crb2a^IntraWT^, the SP from zebrafish Crb2b was introduced with PCR using a pair of primers in which the HA tag sequence was engineered immediately after SP within the reverse primer and the sequence containing the Crb2a transmembrane domain plus the intracellular domain was amplified by PCR and cloned in-framed following the HA sequence. Crb2a^IntraΔPBD ^was made using Crb2a^IntraWT ^as a template and site-directed mutagenesis was performed to introduce a stop codon after the last glutamic acid residue (E16) in the FERM-binding domain. To make Crb2a^IntraΔFBD^, the conserved amino acids tyrosine (T10) and glutamic acid (E16) of Crb2a^IntraWT ^were substituted with alanines to compromise the FERM-binding domain as described by Izaddoost and colleagues [14]. Site-directed mutagenesis was performed on Crb2a^IntraΔPBD ^to introduce a stop codon after E16 to make Crb2a^IntraΔFBDΔPBD^. All constructs contain a poly-adenylation sequence at the 3'-end. The constructs were cloned into pTol [18,19].

### Transgenesis

Transgenic zebrafish lines were generated using pTol system [18,19]. We coinjected 40 ng/mL of pTol-transgene construct plasmid with 40 ng/mL transposase mRNA into one-cell stage Tg(*Xop:EGFP*);*alb*^-/+ ^embryos. Injected embryos were grown to adulthood and out-crossed with Tg(*Xop:EGFP*);*alb*^-/+ ^to produce offspring. We used PCR to identify transgenic offspring; forward primer GGCATGCCGTCCCTAAAAG designed within the promoter region, and the reverse primer AGCGTAATCTGGAACATCGTAT within the HA tag sequence. We identified three germline transgenic founders (F0) for each construct and generated F1 lines. We confirmed transgene expression by anti-HA immunohistochemistry. Transgenic F1s and subsequent generations were identified by PCR on fin clip DNA. F1 carriers were out-crossed with Tg(*Xop*:EGFP);*alb*^-/+ ^line to produce F2s.

### In Vitro GST Interaction

Construction and expression of Crb2a fusion proteins and HIS-Moe_FERM were described previously [8]. GST-Crb2a^IntraΔFBD ^was made using site-directed mutagenesis on GST-Crb2a^IntraWT ^(as described above) to introduce mutations in two residues of FBD (E10 and T16). Protein interactions were performed as described previously [8], using 10 mg of His-Moe_FERM incubated with 10 mg of GST, GST- Crb2a^IntraWT ^or GST-Crb2a^IntraΔFBD^.

### Immunocytochemistry and Microscopy

Production and levels of transgene products were assessed on 6 d retinal sections by anti-HA antibody labeling. We fixed 6 d zebrafish in the afternoon in 4% paraformaldehyde. Cryostat sections (20-30 μm) were treated with 0.1% SDS for 15 min, washed in PBS with 0.1% Tween (PBS-Tw), incubated in 10% goat serum in PBS-Tw, rinsed briefly in PBS-Tw, and incubated overnight at 4°C in primary antibody (monoclonal anti-HA IgG_1_, 1:1000 (Covance); monoclonal anti-HA IgG_3_, 1:500 (Upstate); rabbit anti-Moe, 1:1000; rabbit anti-panCrb (we raised against the synthetic peptide, AGARLEMDSVLKVPPEERLI), 1:500; anti-aPKCζ, 1:1000 (Santa Cruz); rabbit anti-GFP 1:200 (Molecular Probes), anti-Rhodopsin monoclonal R6-5, 1:50 [21]; zs-4 antibody, 1:10 (University of Oregon Monoclonal Antibody Facility); rabbit anti-GOLGA5 1:500 (Sigma HPA000992). Sections were washed, incubated with the appropriate secondary antibodies (-FITC/-TRITC (Molecular Probes) 1:100, -CY5 1:100 (Jackson) goat anti-mouse IgG3 rhodamine red-conjugated, 1:100; goat anti-mouse IgG_1 _Cy-5-conjugated, 1:250; goat anti-mouse IgG_1 _rhodamine red-conjugated, 1:100; goat anti-mouse IgG_2a _Cy-5-conjugated, 1:100 (Jackson Laboratory)), and analyzed with a Zeiss LSM 510 Meta Confocal System. We primary analyzed the retinas in *alb*^-/- ^individuals to ensure that the entire outer segment was visible and not obscured by the RPE; the localization of transgene products was the same in pigmented siblings (data not shown). Confocal images are a single scan (averaged 4 times) at about 1 μm optical thickness. Volume of wild-type and transgenic rods was measured using Sync Measure 3D function of Image J. Only cells that were completely captured in the confocal stacks were measured. Outer segments were outlined and measured by an overlap of anti-GFP and R6-5 labeling; cell compartments that are free of R6-5 labeling were outlined and measured as inner segment plus cell body. The width of inner segment (Fig. 4C) and the area of myoid regions (Fig. 4D) were outlined and measured using Image J.

## Abbreviations

OLM: Outer limiting membrane; FBD: FERM-binding domain; PBD: PDZ-binding domain; SP: signal peptide; TM: transmembrane domain; EXTRA_SECR: Extracellular domain secreted; EXTRA_TM: Extracellular domain transmembrane domain; FL: full-length.

## Authors' contributions

AMJ and YCH conceived of and designed the study. YCH and AMJ performed the experiments and collected the data. AMJ and YCH drafted the manuscript. All authors read and approved the final manuscript.

## Supplementary Material

Additional file 1 **Single confocal z-sections of rods expressing Crb2a transgenes**. (A, G') Transgenic rods at 6 d labeled with GFP antibodies (green) and Rhodopsin antibodies (blue), and anti-HA antibodies (red). (A, A') Crb2a^FL ^transgenic rods. (B, B') Crb2a^Extra_TM ^transgenic rods. (C, C') Crb2a^Extra_Secr ^transgenic rods. (D, D') Crb2a^IntraWT ^transgenic rods. (E, E') Crb2a^IntraΔFBD ^transgenic rods. (F, F') Crb2a^IntraΔPBD ^transgenic rods. (G, G') Crb2a^IntraΔFBDΔPBD ^transgenic rods. Scale bar, 10 μm. Click here for file

Additional file 2 **Crb2a^Extra_Secr ^surrounds outer segments of cones**. Single confocal z-section of a 6 d Crb2a^Extra_Secr ^transgenic retina labeled with zs-4 antibodies (red), rhodopsin antibodies (blue) and anti-GFP antibodies (green) merged with the DIC-like image. Dotted yellow circles indicate the lipid droplet in double-cone inner segments, dotted white line indicates the outer limiting membrane. Scale bar, 5 μm. Click here for file

Additional file 3 **The Golgi apparatus, labeled by antibodies to Golgin subfamily A member 5 (GOLGA5), in 6 d wild-type and Crb2a transgenics**. (A-G"') Confocal z-projection of GFP-expressing rods in sections of 6 d retinas labeled with anti-GOLGA5 antibodies (red) and anti-HA (blue). (A, A') Wild-type rods. (B-B"') Crb2a^FL ^transgenic rods. (C-C"') Crb2a^Extra_TM ^transgenic rods. (D-D"') Crb2a^Extra_Secr ^transgenic rods. (E-E"') Crb2a^IntraWT ^transgenic rods. (F-F"') Crb2a^IntraΔFBD ^transgenic rods. (G-G"') Crb2a^IntraΔPBD ^transgenic rods. (H) Western blot of 5 d wild-type zebrafish labeled with anti-GOLGA5 antibodies reveals a single protein of the expected molecular weight. The western blot was performed as described in [8], anti-GOLGA5 (Sigma HPA000992) was used at 1:1000 and HRP-conjugated goat anti-rabbit was used at 1:30,000. Scale bar, 5 μm. Click here for file

Additional file 4 **Effects of Crb2a construct expression on Moe localization**. (A-H") Confocal z-projection of 6 d photoreceptor layer labeled with anti-GFP (green), anti-HA antibodies (red) and anti-Moe antibodies (blue). (A-A') Wild-type rods. (B-B") Crb2a^IntraWT ^transgenic rods. (C-C") Crb2a^IntraΔFBD ^transgenic rods. (D-D") Crb2a^IntraΔPBD ^transgenic rods. (E-E") Crb2a^IntraΔFBDΔPBD ^transgenic rods. (F-F") Crb2a^FL ^transgenic rods. (G-G") Crb2a^Extra_TM ^transgenic rods. (H-H") Crb2a^Extra_Secr ^transgenic rods. Scale bars, 5 μm. Click here for file

Additional file 5 **Effects of Crb2a construct expression on Prkci localization**. (A-H"') Confocal z-projection of 6 d photoreceptor layer labeled with anti-HA (red), anti-Prkci antibodies (blue) and anti-GFP labeling (green). (A-A") Wild-type photoreceptor rods. (B-B"') Crb2a^IntraWT ^transgenic rods. (C-C"') Crb2a^IntraΔFBD ^transgenic rods. (D-D"') Crb2a^IntraΔPBD ^transgenic rods. (E-E"') Crb2a^IntraΔFBDΔPBD ^transgenic rods. (F-F"') Crb2a^FL ^transgenic rods. (G-G"') Crb2a^Extra_TM ^transgenic rods. (H-H"') Crb2a^Extra_Secr ^transgenic rods. Scale bars, 5 μm. Click here for file

Additional file 6 **Western blot of 6 d wild-type (nonTg WT), Crb2a^IntraWT^, Crb2a^IntraΔFBD^, Crb2a^IntraΔPBD^, Crb2a^IntraΔFBDΔPBD^, Crb2a^FL^, Crb2a^Extra_TM^, Crb2a^Extra_Secr ^transgenics probed with anti-HA antibodies**. Western blotting was performed as previously described [8] and probed with anti-HA (clone 16B12) and HRP-conjugated goat anti-mouse. The predicted molecular weight of Crb2a^IntraWT ^protein is 11 kD (retaining the signal peptide) but on western blot the major Crb2a^IntraWT ^protein is ~30kD, suggesting that it may be post-translationally modified or forms homoligomeres. Despite trying multiple gel and transfer conditions we were unable to detect Crb2a^IntraΔFBD^, Crb2a^IntraΔPBD^, Crb2a^IntraΔFBDΔPBD ^proteins, which by immunohistochemistry are expressed at similar levels as Crb2a^IntraWT^. It is possible that Crb2a^IntraΔFBD^, which is be predicted to be about the same molecular weight as Crb2a^IntraWT^, is not post-translationally modified or does not dimerize and, thus, is too small, like Crb2a^IntraΔPBD ^and Crb2a^IntraΔFBDΔPBD ^with predicted molecular weights ~8.8kD (with signal peptide) to be captured by Western blot analysis. Click here for file

## References

[B1] Young RW Bok D Participation of the retinal pigment epithelium in the rod outer segment renewal process J Cell Bio 1969 42 392 403 10.1083/jcb.42.2.392 PMC2107669 5792328

[B2] Anderson DH Fisher SK Disc shedding in rodlike and conelike photoreceptors of tree squirrels Science 1975 187 953 955 10.1126/science.1145180 1145180

[B3] Young RW The renewal of photoreceptor cell outer segments J Cell Bio 1967 33 61 72 10.1083/jcb.33.1.61 PMC2107286 6033942

[B4] Wodarz A Hinz U Engelbert M Knust E Expression of crumbs confers apical character on plasma membrane domains of ectodermal epithelia of Drosophila Cell 1995 82 67 76 10.1016/0092-8674(95)90053-5 7606787

[B5] Pellikka M Tanentzapf G Pinto M Smith C McGlade CJ Ready DF Tepass U Crumbs, the Drosophila homologue of human CRB1/RP12, is essential for photoreceptor morphogenesis Nature 2002 416 143 149 10.1038/nature721 11850625

[B6] Omori Y Malicki J *oko meduzy *and related *crumbs *genes are determinants of apical cell features in the vertebrate embryo Curr Biol 2006 16 945 957 10.1016/j.cub.2006.03.058 16713951

[B7] Jensen AM Westerfield M Zebrafish mosaic eyes is a novel FERM protein required for retinal lamination and retinal pigmented epithelial tight junction formation Curr Biol 2004 14 711 717 10.1016/j.cub.2004.04.006 15084287

[B8] Hsu YC Willoughby JJ Christensen AK Jensen AM Mosaic Eyes is a Novel Component of the Crumbs Complex and Negatively Regulates Photoreceptor Apical Size Development 2006 133 4849 4859 10.1242/dev.02685 17092952 PMC2836018

[B9] den Hollander AI ten Brink JB de Kok YJ van Soest S van den Born LI van Driel MA van de Pol DJ Payne AM Bhattacharya SS Kellner U Hoyng CB Westerveld A Brunner HG Bleeker-Wagemakers EM Deutman AF Heckenlively JR Cremers FP Bergen AA Mutations in a human homologue of Drosophila crumbs cause retinitis pigmentosa (RP12) Nat Genet 1999 23 217 221 10.1038/13848 10508521

[B10] den Hollander AI Heckenlively JR van den Born LI de Kok YJ van der Velde-Visser SD Kellner U Jurklies B van Schooneveld MJ Blankenagel A Rohrschneider K Wissinger B Cruysberg JR Deutman AF Brunner HG Apfelstedt-Sylla E Hoyng CB Cremers FP Leber congenital amaurosis and retinitis pigmentosa with Coats-like exudative vasculopathy are associated with mutations in the crumbs homologue 1 (CRB1) gene Am J Hum Genet 2001 69 198 203 10.1086/321263 11389483 PMC1226034

[B11] Lotery AJ Jacobson SG Fishman GA Weleber RG Fulton AB Namperumalsamy P Heon E Levin AV Grover S Rosenow JR Kopp KK Sheffield VC Stone EM Mutations in the CRB1 gene cause Leber congenital amaurosis Arch Ophthalmol 2001 119 415 420 11231775 10.1001/archopht.119.3.415

[B12] Gerber S Perrault I Hanein S Shalev S Zlotogora J Barbet F Ducroq D Dufier J Munnich A Rozet J Kaplan J A novel mutation disrupting the cytoplasmic domain of CRB1 in a large consanguineous family of Palestinian origin affected with Leber congenital amaurosis Ophthalmic Genet 2002 23 225 235 10.1076/opge.23.4.225.13879 12567265

[B13] den Hollander AI Davis J van der Velde-Visser SD Zonneveld MN Pierrottet CO Koenekoop RK Kellner U van den Born LI Heckenlively JR Hoyng CB Handford PA Roepman R Cremers FP CRB1 mutation spectrum in inherited retinal dystrophies Hum Mutat 2004 24 355 69 10.1002/humu.20093 15459956

[B14] Izaddoost S Nam SC Bhat MA Bellen HJ Choi KW Crumbs is a positional cue in photoreceptor adherens junctions and rhabdomeres Nature 2002 416 178 183 10.1038/nature720 11850624

[B15] Johnson K Grawe F Grzeschik N Knust E Drosophila crumbs is required to inhibit light-induced photoreceptor degeneration Curr Biol 2002 12 1675 1680 10.1016/S0960-9822(02)01180-6 12361571

[B16] Tepass U Theres C Knust E crumbs encodes an EGF-like protein expressed on apical membranes of Drosophila epithelial cells and required for organization of epithelia Cell 1990 61 787 799 10.1016/0092-8674(90)90189-L 2344615

[B17] Mani SS Batni S Whitaker L Chen S Engbretson G Knox BE Xenopus rhodopsin promoter. Identification of immediate upstream sequences necessary for high level, rod-specific transcription J Biol Chem 2001 276 36557 36565 10.1074/jbc.M101685200 11333267

[B18] Kawakami K Shima A Kawakami N Identification of a functional transposase of the Tol2 element, an Ac-like element from the Japanese medaka fish, and its transposition in the zebrafish germ lineage Proc Natl Acad Sci USA 2000 97 11403 8 10.1073/pnas.97.21.11403 11027340 PMC17212

[B19] Kawakami K Takeda H Kawakami N Kobayashi M Matsuda N Mishina M A transposon-mediated gene trap approach identifies developmentally regulated genes in zebrafish Dev Cell 2004 7 133 144 10.1016/j.devcel.2004.06.005 15239961

[B20] Fadool JM Development of a rod photoreceptor mosaic revealed in transgenic zebrafish Dev Biol 2003 258 277 290 10.1016/S0012-1606(03)00125-8 12798288

[B21] Rohlich P Adamus G HcDowell JH Hargrave PA Binding pattern of anti-rhodopsin monoclonal antibodies to photoreceptor cells: an immunocytochemical study Exp Eye Res 1989 49 999 1013 10.1016/S0014-4835(89)80022-3 2612590

[B22] Wodarz A Ramrath A Grimm A Knust E Drosophila atypical protein kinase C associates with Bazooka and controls polarity of epithelia and neuroblasts Cell Biol 2000 150 1361 1374 10.1083/jcb.150.6.1361 PMC2150710 10995441

[B23] Hurd TW Gao L Roh MH Macara IG Margolis B Direct interaction of two polarity complexes implicated in epithelial tight junction assembly Nat Cell 2003 5 137 142 10.1038/ncb923 12545177

[B24] Sotillos S Diaz-Meco MT Caminero E Moscat J Campuzano S DaPKC-dependent phosphorylation of Crumbs is required for epithelial cell polarity in Drosophila J Cell Biol 2004 166 549 557 10.1083/jcb.200311031 15302858 PMC2172211

[B25] den Hollander AI Ghiani M de Kok YJ Wijnholds J Ballabio A Cremers FP Broccoli V Isolation of Crb1, a mouse homologue of Drosophila crumbs, and analysis of its expression pattern in eye and brain Mech Dev 2002 110 203 207 10.1016/S0925-4773(01)00568-8 11744384

[B26] van den Hurk JA Rashbass P Roepman R Davis J Voesenek KE Arends ML Zonneveld MN van Roeke MH Cameron K Rohrschneider K Heckenlively JR Koenekoop RK Hoyng CB Cremers FP den Hollander AI Characterization of the Crumbs homologue 2 (CRB2) gene and analysis of its role in retinitis pigmentosa and Leber congenital amaurosis Mol Vis 2005 11 263 273 15851977

[B27] Richard M Roepman R Aartsen WM van Rossum AGSH den Hollander AI Knust E Wijnholds J Cremers FPM Towards understanding CRUMBS function in retinal dystrophies Human Mol Genet 2006 15 R235 R243 10.1093/hmg/ddl195 16987889

[B28] Mehalow AK Kameya S Smith RS Hawes NL Denegre JM Young JA Bechtold L Haider NB Tepass U Heckenlively JR Chang B Naggert JK Nishina PM CRB1 is essential for external limiting membrane integrity and photoreceptor morphogenesis in the mammalian retina Hum Mol Genet 2003 12 2179 2189 10.1093/hmg/ddg232 12915475

[B29] van de Pavert SA Kantardzhieva A Malysheva A Meuleman J Versteeg I Levelt C Klooster J Geiger S Seeliger MW Rashbass P Le Bivic A Wijnholds J Crumbs homologue 1 is required for maintenance of photoreceptor cell polarization and adhesion during light exposure J Cell Sci 2004 117 4169 4177 10.1242/jcs.01301 15316081

[B30] Christensen AK Jensen AM Tissue-specific requirements for specific domains in the FERM protein Moe/Epb4.1l5 during early zebrafish development BMC Dev Biol 2008 8 3 10.1186/1471-213X-8-3 18190700 PMC2266719

[B31] Kantardzhieva A Gosens I Alexeeva S Punte IM Versteeg I Krieger E Neefjes-Mol CA den Hollander AI Letteboer SJ Klooster J Cremmers FP Roepman R Wijnholds J MPP5 recruits MPP4 to the CRB1 complex in photoreceptors Invest Ophthalmol Vis Sci 2005 46 2192 2201 10.1167/iovs.04-1417 15914641

[B32] van Rossum AG Aartsen WM Meuleman J Klooster J Malysheva A Versteeg I Arsanto JP Le Bivic A Wijnholds J Pals1/Mpp5 is required for correct localization of Crb1 at the subapical region in polarized Muller glia cells Hum Mol Genet 2006 15 2659 72 10.1093/hmg/ddl194 16885194

[B33] Gosens I van Wijk E Kersten FFJ Krieger E van der Zwaag B Märker T Letteboer SJF Dusseljee S Peters T Spierenburg HA Punte IM Wolfrum U Cremers FP Kremer H Roepman R MPP1 links the Usher protein network and the Crumbs protein complex in the retina Human Mol Genet 2007 16 1993 2003 10.1093/hmg/ddm147 17584769

[B34] Baker SA Haeri M Yoo P Gospe SM Skiba NP Knox BE Arshavsky VY The outer segment serves as a default destination for the trafficking of membrane proteins in photoreceptors J Cell Biol 2008 183 485 98 10.1083/jcb.200806009 18981232 PMC2575789

[B35] Pichaud F Desplan C Cell biology: a new view of photoreceptors Nature 2002 416 139 40 10.1038/416139a 11894082

[B36] Laprise P Beronja S Silva-Gagliardi N Pellikka M Jensen AM McGlade J Tepass U The FERM Protein Yurt is a Component of the Crumbs Complex and Negatively Regulates Apical Membrane Size Dev Cell 2006 11 363 374 10.1016/j.devcel.2006.06.001 16950127 PMC2834949

[B37] Klebes A Knust E A conserved motif in Crumbs is required for E-cadherin localization and zonula adherens formation in *Drosophila* Curr Biol 2000 10 76 85 10.1016/S0960-9822(99)00277-8 10662667

[B38] Westerfield M The Zebrafish Book 1995 University of Oregon Press, Eugene

