# Supplementary figures and images for: Multiple domains in the Crumbs Homolog 2a (Crb2a) protein are required for regulating rod photoreceptor size
Source: BMC Cell Biol. 2010 Jul 29;11:60. doi: 10.1186/1471-2121-11-60 (PMC2927502; doi:10.1186/1471-2121-11-60)

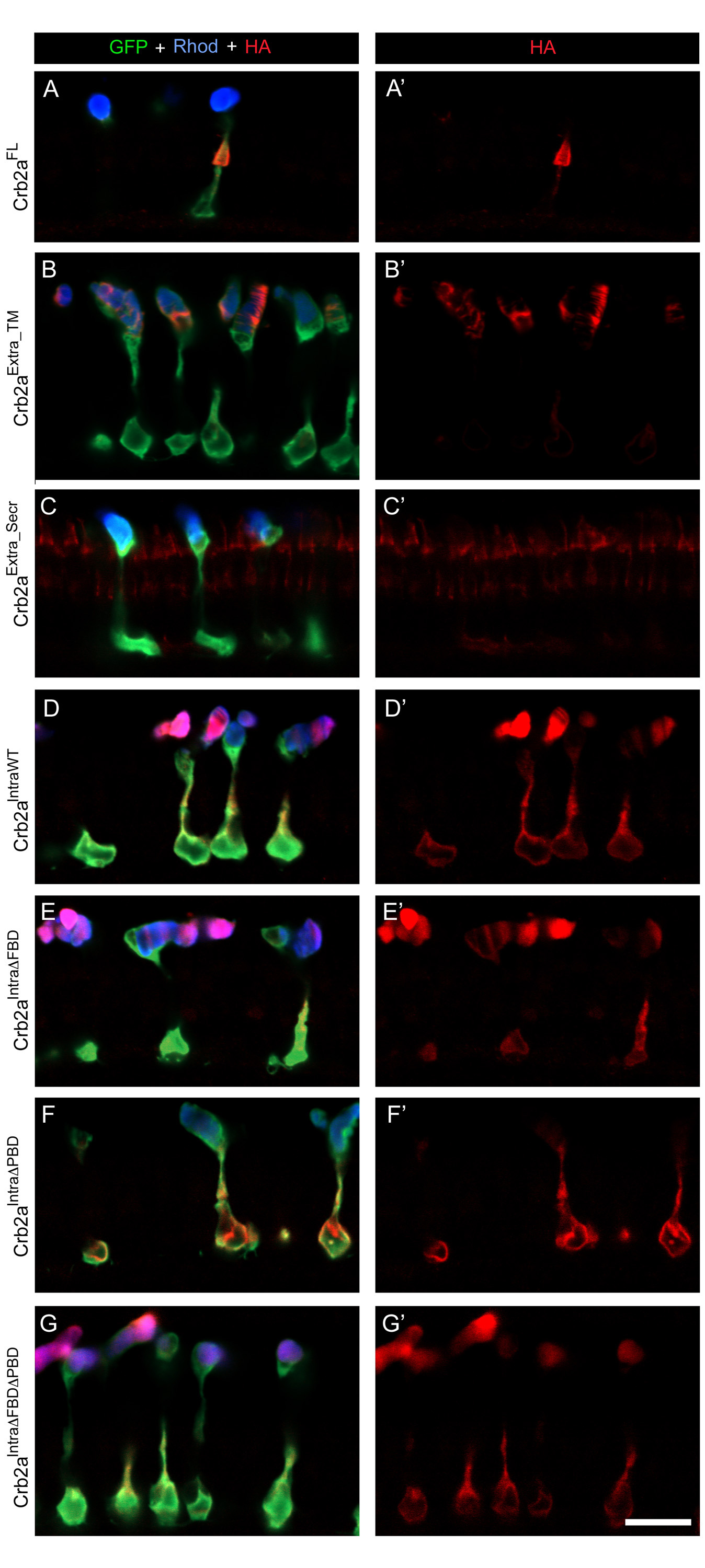

Supplement: Additional file 1 — Single confocal z-sections of rods expressing Crb2a transgenes. (A, G') Transgenic rods at 6 d labeled with GFP antibodies (green) and Rhodopsin antibodies (blue), and anti-HA antibodies (red). (A, A') Crb2aFL transgenic rods. (B, B') Crb2aExtra_TM transgenic rods. (C, C') Crb2aExtra_Secr transgenic rods. (D, D') Crb2aIntraWT transgenic rods. (E, E') Crb2aIntraΔFBD transgenic rods. (F, F') Crb2aIntraΔPBD transgenic rods. (G, G') Crb2aIntraΔFBDΔPBD transgenic rods. Scale bar, 10 μm. [file 1471-2121-11-60-S1.JPEG]

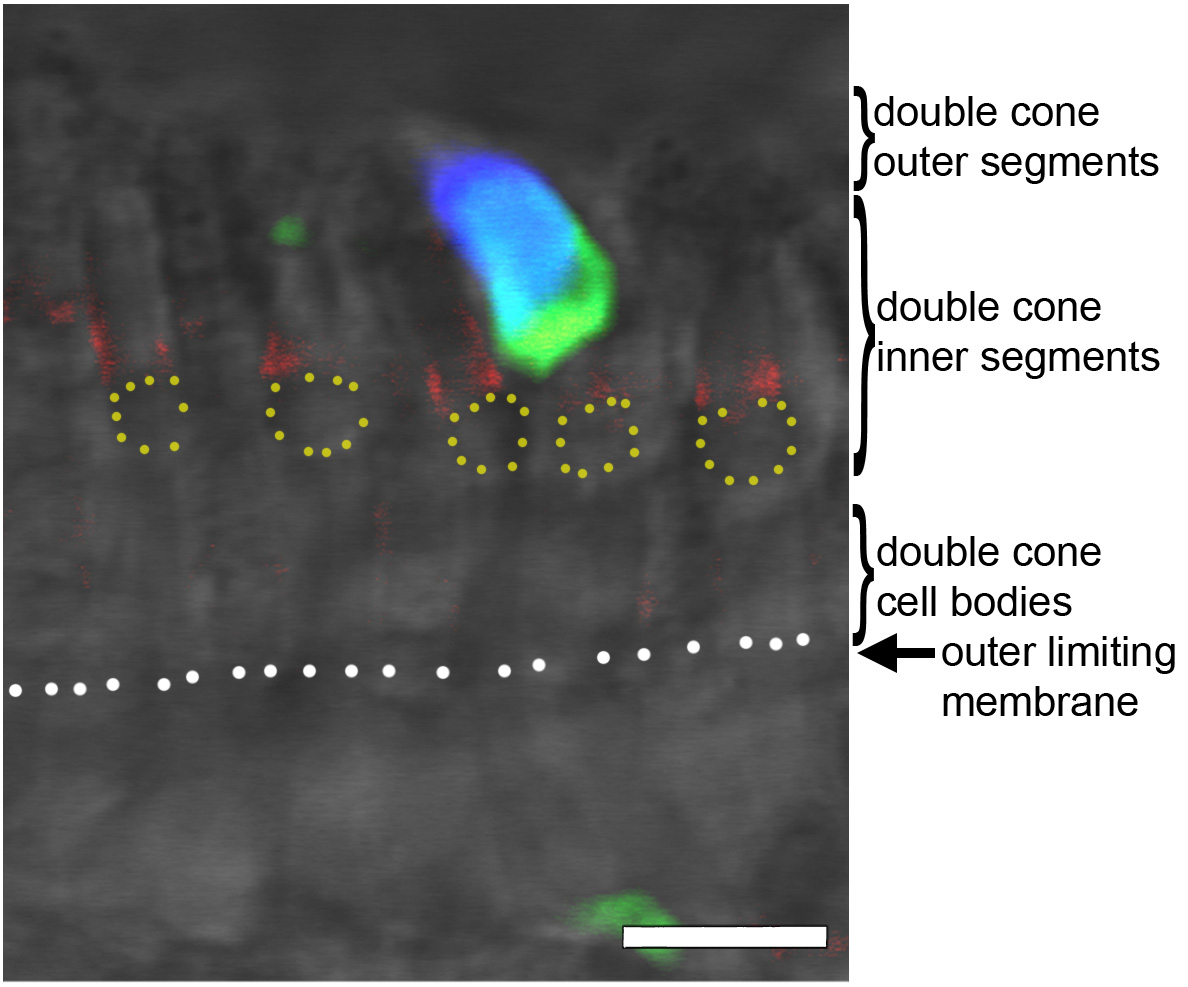

Supplement: Additional file 2 — Crb2aExtra_Secr surrounds outer segments of cones. Single confocal z-section of a 6 d Crb2aExtra_Secr transgenic retina labeled with zs-4 antibodies (red), rhodopsin antibodies (blue) and anti-GFP antibodies (green) merged with the DIC-like image. Dotted yellow circles indicate the lipid droplet in double-cone inner segments, dotted white line indicates the outer limiting membrane. Scale bar, 5 μm. [file 1471-2121-11-60-S2.JPEG]

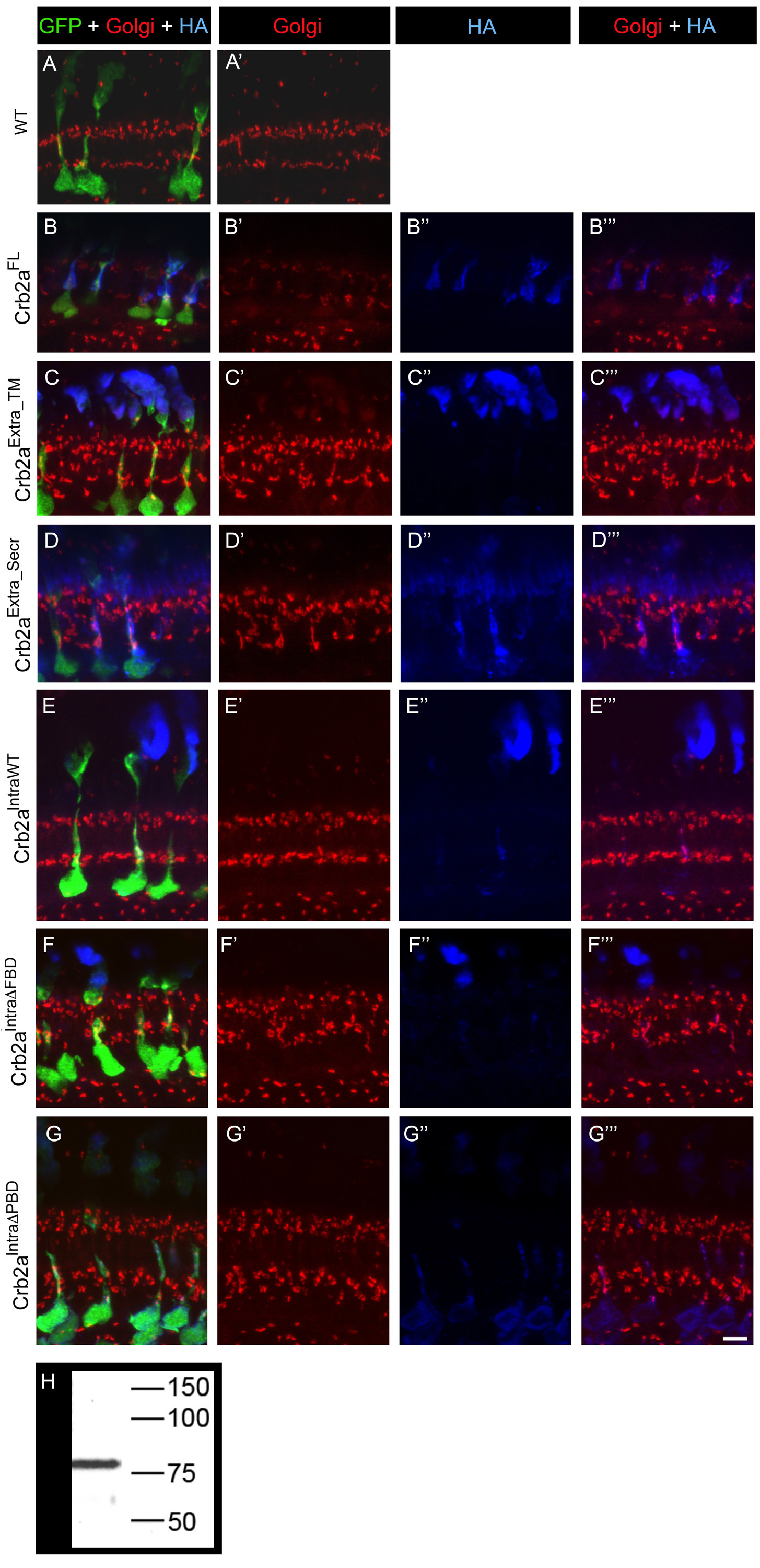

Supplement: Additional file 3 — The Golgi apparatus, labeled by antibodies to Golgin subfamily A member 5 (GOLGA5), in 6 d wild-type and Crb2a transgenics. (A-G"') Confocal z-projection of GFP-expressing rods in sections of 6 d retinas labeled with anti-GOLGA5 antibodies (red) and anti-HA (blue). (A, A') Wild-type rods. (B-B"') Crb2aFL transgenic rods. (C-C"') Crb2aExtra_TM transgenic rods. (D-D"') Crb2aExtra_Secr transgenic rods. (E-E"') Crb2aIntraWT transgenic rods. (F-F"') Crb2aIntraΔFBD transgenic rods. (G-G"') Crb2aIntraΔPBD transgenic rods. (H) Western blot of 5 d wild-type zebrafish labeled with anti-GOLGA5 antibodies reveals a single protein of the expected molecular weight. The western blot was performed as described in [8], anti-GOLGA5 (Sigma HPA000992) was used at 1:1000 and HRP-conjugated goat anti-rabbit was used at 1:30,000. Scale bar, 5 μm. [file 1471-2121-11-60-S3.JPEG]

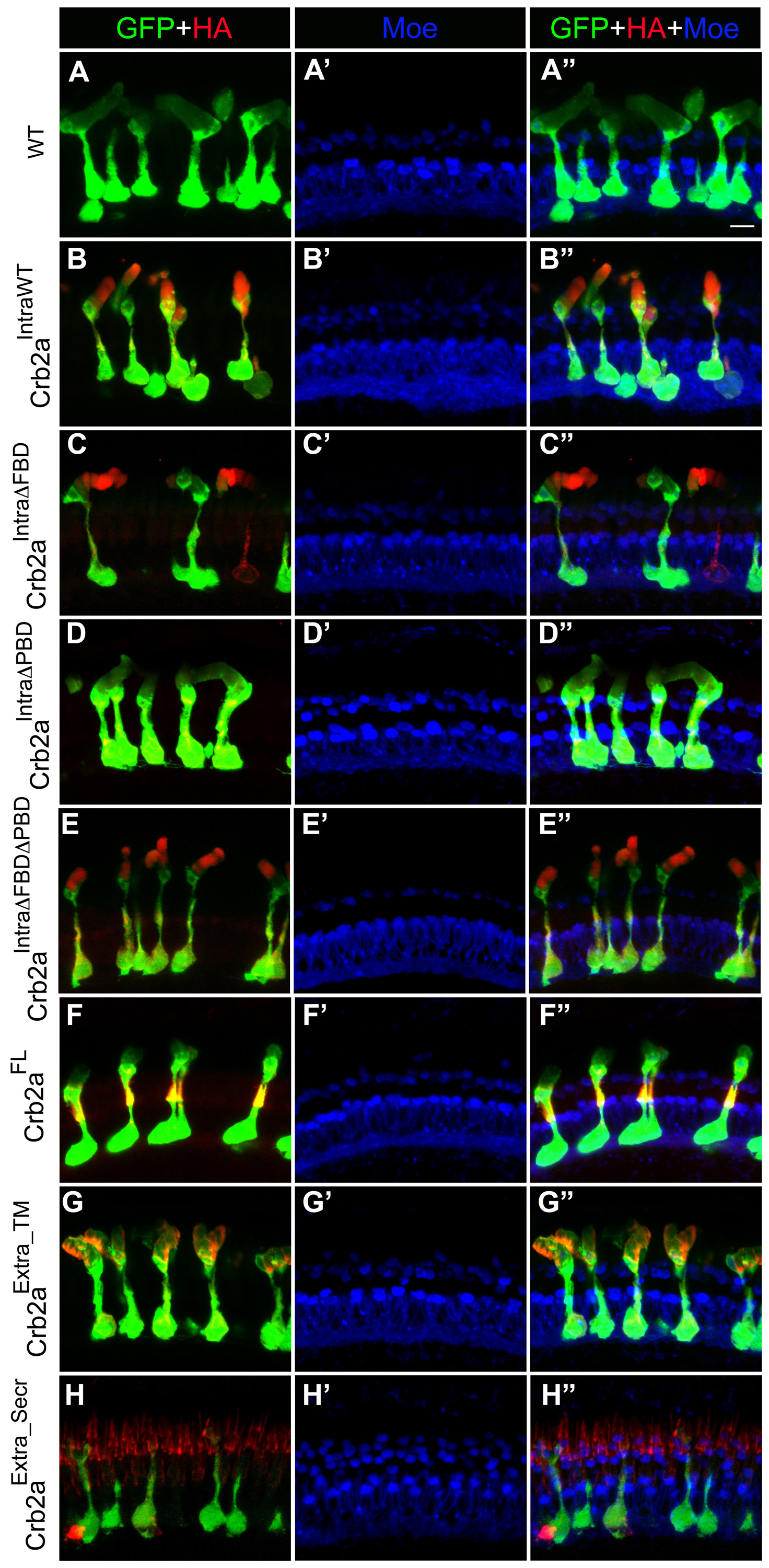

Supplement: Additional file 4 — Effects of Crb2a construct expression on Moe localization. (A-H") Confocal z-projection of 6 d photoreceptor layer labeled with anti-GFP (green), anti-HA antibodies (red) and anti-Moe antibodies (blue). (A-A') Wild-type rods. (B-B") Crb2aIntraWT transgenic rods. (C-C") Crb2aIntraΔFBD transgenic rods. (D-D") Crb2aIntraΔPBD transgenic rods. (E-E") Crb2aIntraΔFBDΔPBD transgenic rods. (F-F") Crb2aFL transgenic rods. (G-G") Crb2aExtra_TM transgenic rods. (H-H") Crb2aExtra_Secr transgenic rods. Scale bars, 5 μm. [file 1471-2121-11-60-S4.JPEG]

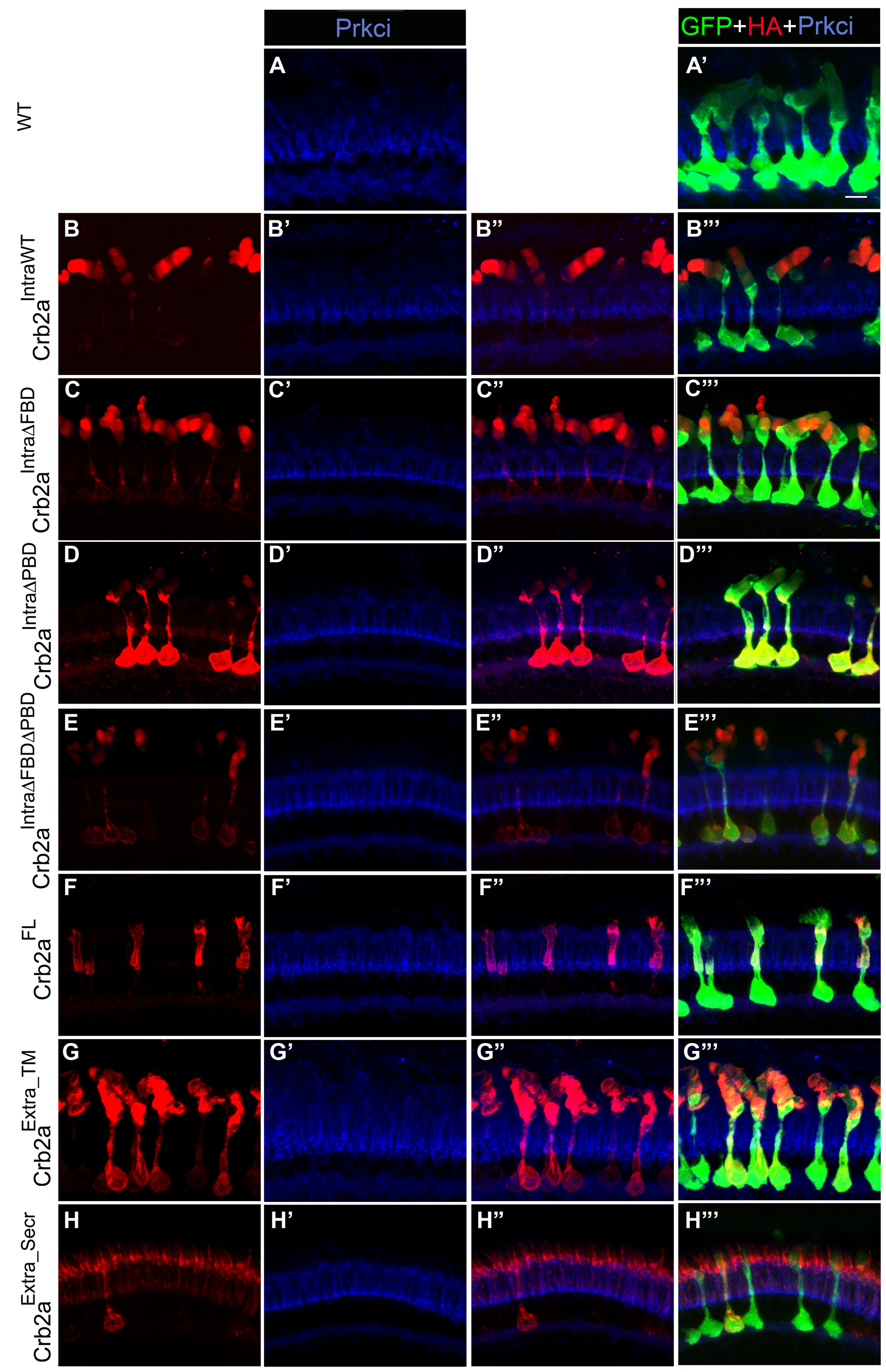

Supplement: Additional file 5 — Effects of Crb2a construct expression on Prkci localization. (A-H"') Confocal z-projection of 6 d photoreceptor layer labeled with anti-HA (red), anti-Prkci antibodies (blue) and anti-GFP labeling (green). (A-A") Wild-type photoreceptor rods. (B-B"') Crb2aIntraWT transgenic rods. (C-C"') Crb2aIntraΔFBD transgenic rods. (D-D"') Crb2aIntraΔPBD transgenic rods. (E-E"') Crb2aIntraΔFBDΔPBD transgenic rods. (F-F"') Crb2aFL transgenic rods. (G-G"') Crb2aExtra_TM transgenic rods. (H-H"') Crb2aExtra_Secr transgenic rods. Scale bars, 5 μm. [file 1471-2121-11-60-S5.JPEG]

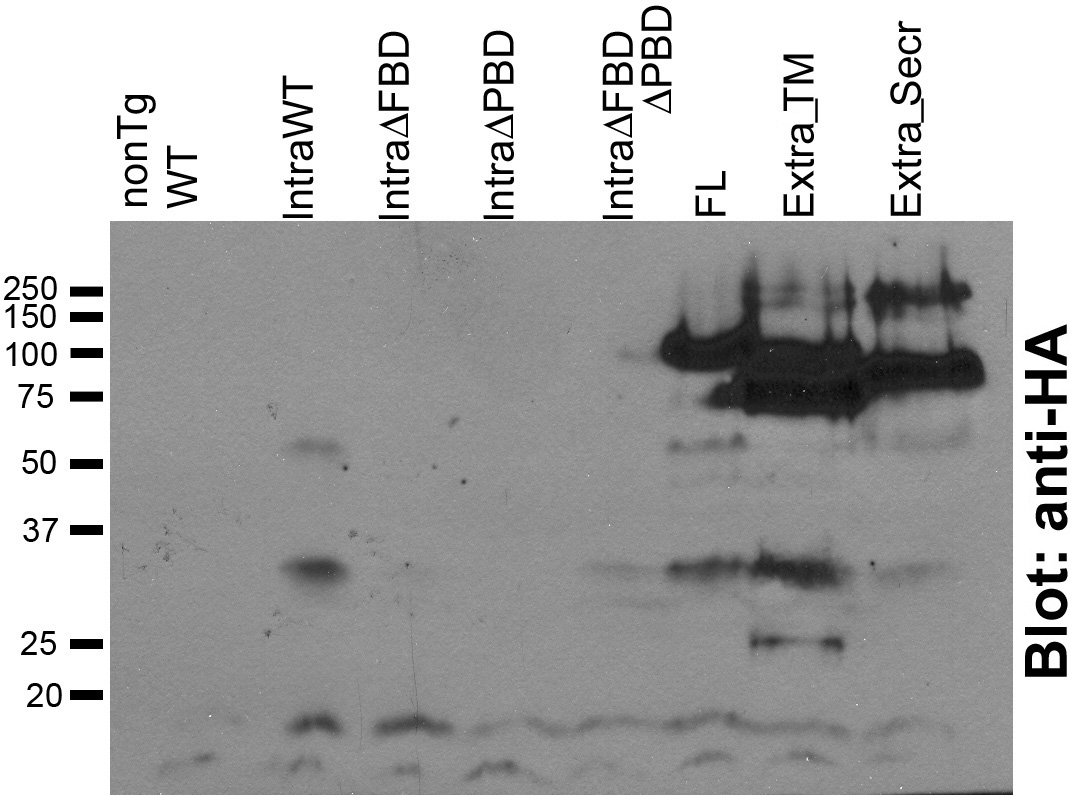

Supplement: Additional file 6 — Western blot of 6 d wild-type (nonTg WT), Crb2aIntraWT, Crb2aIntraΔFBD, Crb2aIntraΔPBD, Crb2aIntraΔFBDΔPBD, Crb2aFL, Crb2aExtra_TM, Crb2aExtra_Secr transgenics probed with anti-HA antibodies. Western blotting was performed as previously described [8] and probed with anti-HA (clone 16B12) and HRP-conjugated goat anti-mouse. The predicted molecular weight of Crb2aIntraWT protein is 11 kD (retaining the signal peptide) but on western blot the major Crb2aIntraWT protein is ~30kD, suggesting that it may be post-translationally modified or forms homoligomeres. Despite trying multiple gel and transfer conditions we were unable to detect Crb2aIntraΔFBD, Crb2aIntraΔPBD, Crb2aIntraΔFBDΔPBD proteins, which by immunohistochemistry are expressed at similar levels as Crb2aIntraWT. It is possible that Crb2aIntraΔFBD, which is be predicted to be about the same molecular weight as Crb2aIntraWT, is not post-translationally modified or does not dimerize and, thus, is too small, like Crb2aIntraΔPBD and Crb2aIntraΔFBDΔPBD with predicted molecular weights ~8.8kD (with signal peptide) to be captured by Western blot analysis. [file 1471-2121-11-60-S6.JPEG]
